# Protocol of the LEONORA randomized clinical trial: Lower gastrointestinal symptom burden by prophylaxis with synbiotics after colorectal cancer surgery

**DOI:** 10.1186/s12885-026-15903-9

**Published:** 2026-03-25

**Authors:** Ben Schöttker, Annette Kopp-Schneider, Lena Biehl

**Affiliations:** 1 Division of Clinical Epidemiology of Early Cancer Detection, German Cancer Research Center (DKFZ), Im Neuenheimer Feld 581, Heidelberg, 69120 Germany; 2 Division of Biostatistics, German Cancer Research Center (DKFZ), Im Neuenheimer Feld 581, Heidelberg, 69120 Germany; 3 Department I of Internal Medicine, University Hospital of Cologne, Cologne, Kerpener Str. 62 50937 Germany; 4 German Centre of Infection Research (partner site Bonn-Cologne), Cologne, Germany; 5 Fraunhofer Institute for Translational Medicine and Pharmacology ITMP and Fraunhofer Cluster of Excellence Immune-Mediated Diseases CIMD, Frankfurt am Main, Germany

**Keywords:** Randomized controlled trial; synbiotics, Inulin, Colorectal cancer, Quality of life, Gut microbiome, Infections, Inflammation, Metabolites

## Abstract

**Background:**

A key component of colorectal cancer (CRC) treatment is the surgical resection of the tumor, accompanied by antibiotic treatment and often chemotherapy, which severely alter the gut function and its microbiome. Thus, CRC patients commonly suffer from gastrointestinal symptoms like chronic diarrhea, which can persist in some patients for several years.

Synbiotics are a mixture comprising live microorganisms and substrate(s) selectively utilized by host microorganisms that confer a health benefit on the host. Previous randomized controlled trials have shown that synbiotics reduce rates of post-operative infections and related complications, diarrhea incidence, and inflammation. However, data on the gastrointestinal quality of life are very sparse.

**Methods:**

The proposed LEONORA trial is a randomized controlled trial with 206 CRC patients, who shall be recruited in 10 German clinics. While 103 patients in arm 1 will receive a placebo, 103 patients in arm 2 will be given a daily synbiotic treatment for 12 weeks with 50 billion colony-forming units of 12 live bacterial strains. After the wound healing phase, patients in the intervention arm will additionally receive the dietary fiber inulin from weeks 5 to 12. Every 2 weeks, the inulin dosage will be stepwise increased from 3 g to 10 g per day to slowly train the gut microbiome and avoid flatulence. The study participants in the placebo group, on the other hand, receive maltodextrin as a second placebo, which is very similar to inulin in appearance and taste, but does not affect the gut microbiome.

The primary endpoint is the gastrointestinal quality of life 90 days after surgery. Further outcomes include rates of infections, cancer recurrence, cancer survival, blood-based inflammatory biomarkers, and metabolites. Stool samples will be collected to additionally address changes in the gut microbiome and metabolome composition.

**Discussion:**

The 3-month therapy with synbiotics could be a well-tolerated and very cost-effective approach to increase the gastrointestinal quality of life of CRC patients. Thus, the results of the proposed LEONORA trial will be of high relevance for both caregivers and CRC patients.

**Trial registration:**

German Clinical Trials Register (DRKS): DRKS00034919, November 21, 2024; https://www.drks.de/search/de/trial/DRKS00034919.

**Supplementary Information:**

The online version contains supplementary material available at 10.1186/s12885-026-15903-9.

## Background

The key components of most colorectal cancer (CRC) treatments are the tumor resection, accompanied by perioperative antibiotic prophylaxis. Furthermore, the surgery is followed by adjuvant chemotherapy for specific subgroups of CRC patients. Following CRC resection, disorders in the gastrointestinal tract are often believed to be a temporary issue for about 3–6 months after surgery. However, a substantial proportion of patients still reports symptoms 1 year after their cancer diagnosis with respect to pain (43% of patients), constipation (31%), and diarrhea (31%) [1]. Moreover, chronic diarrhea has been reported by 13–50% of CRC survivors up to 10 years after treatment [2]. In comparison to the general population, CRC survivors more often report suffering from impaired physical quality of life, with bowel problems being very common [3].

Synbiotics can be defined as “a mixture comprising live microorganisms and substrate(s) selectively utilized by host microorganisms that confers a health benefit on the host” [4]. The live microorganisms, also called “probiotics”, can beneficially affect the host by improving the composition and the equilibrium of the bowel microbiota [4]. The substrates, also called “prebiotics”, are non-digestible fibers that reach the colon and serve as a substrate for fermentation by probiotics and indigenous colonic bacteria, providing nutrients important for host health [4]. Synbiotics were found to be more effective than the administration of probiotics alone [5]. Current literature provides no evidence of an increased risk of complications with probiotics supplementation in humans [6].

Preliminary evidence from cell culture, animal studies, and first observational human studies suggests that probiotics and prebiotics have anti-carcinogenic and potentially cancer therapy-enhancing effects [7–9]. Moreover, an increasing number of studies suggest an association between the intestinal microbiota composition and oncogenesis and response to anti-cancer treatment [10]. It has been observed that the efficacy of certain anticancer agents depends on the prevalence of distinct bacterial groups in the gut microbiome, which may be negatively affected by antibiotics [8].

Recently published meta-analyses, summarizing the effects of 14–19 randomized controlled trials (RCTs) with pro- or synbiotics administration in CRC patients, consistently concluded that such treatments reduce post-operative infections, related complications, and the incidence of diarrhoea [11–15]. They led to a faster return to a normal gut function, shortened postoperative antibiotics use, lowered the incidence of sepsis, and shortened the length of hospital stay [11, 12]. Furthermore, anti-inflammatory effects have been documented by decreased C-reactive protein and interleukin-6 levels [16].

In order to avoid conducting a trial that was already done, we searched MEDLINE, Cochrane Central, the Cochrane Library, and clinicaltrialsregister.eu, DRKS, clinicaltrials.gov, and ICTRP on September 25, 2023, for RCTs aiming to demonstrate the effects of probiotic or synbiotic therapy on the quality of life of CRC patients. The trial of Theodoropoulos et al. [17] is the only previous or registered trial that tested/tests the efficacy of synbiotics on the gastrointestinal quality of life among CRC patients shortly after tumor surgery. The LEONORA trial will extend the evidence from the study of Theodoropoulos et al. [17] by the following aspects:

1. More diverse probiotic formulation (7 Lactobacillus, 4 Bifidobacterium, and 1 Streptococcus bacterial strain vs. 4 Lactobacillus bacterial strains).
2. More potent prebiotic (10 g inulin vs. a mix of 2.5 g of inulin with 7.5 g of less potent fibers (β-glucan, pectin, and resistant starch) [18].
3. Longer therapy (90 vs. 15 days).
4. Multi-center approach embedded in the German routine clinical setting instead of a single-center study from Greece.
5. A much larger sample size (*n* = 206 instead of *n* = 67).

With 206 patients, the LEONORA trial would be the largest placebo-controlled RCT worldwide to be conducted on the efficacy of synbiotics in the prevention of postoperative complications in CRC patients. Moreover, the LEONORA trial would be the first on this topic from Germany specific for CRC patients. The only 3 previous studies from Germany included pancreatic cancer patients or heterogeneous cancer populations [19–21]. In addition, it would be the first trial worldwide looking into the effects of synbiotics on the emergence of multidrug-resistant organisms. Furthermore, stool samples will be collected to ascertain changes in microbiota composition. Due to the low case numbers and short follow-up times, all meta-analyses on cancer survival were inconclusive [11–15]. Thus, having another large trial with several years of mortality follow-up time might lead to a statistically significant pooled effect estimate for cancer survival in future meta-analyses.

### Aims of the study

The main objective of this study is to test whether the early post-surgical administration of synbiotics has an impact on the gastrointestinal function-related quality of life 90 days after surgery. Further objectives are to test the efficacy of the intervention on rates of infection, inflammatory biomarkers, and recurrence-free survival. Stool samples will be collected to additionally address changes in microbiota composition.

## Methods/Design

### Study design

**Fig. 1:**
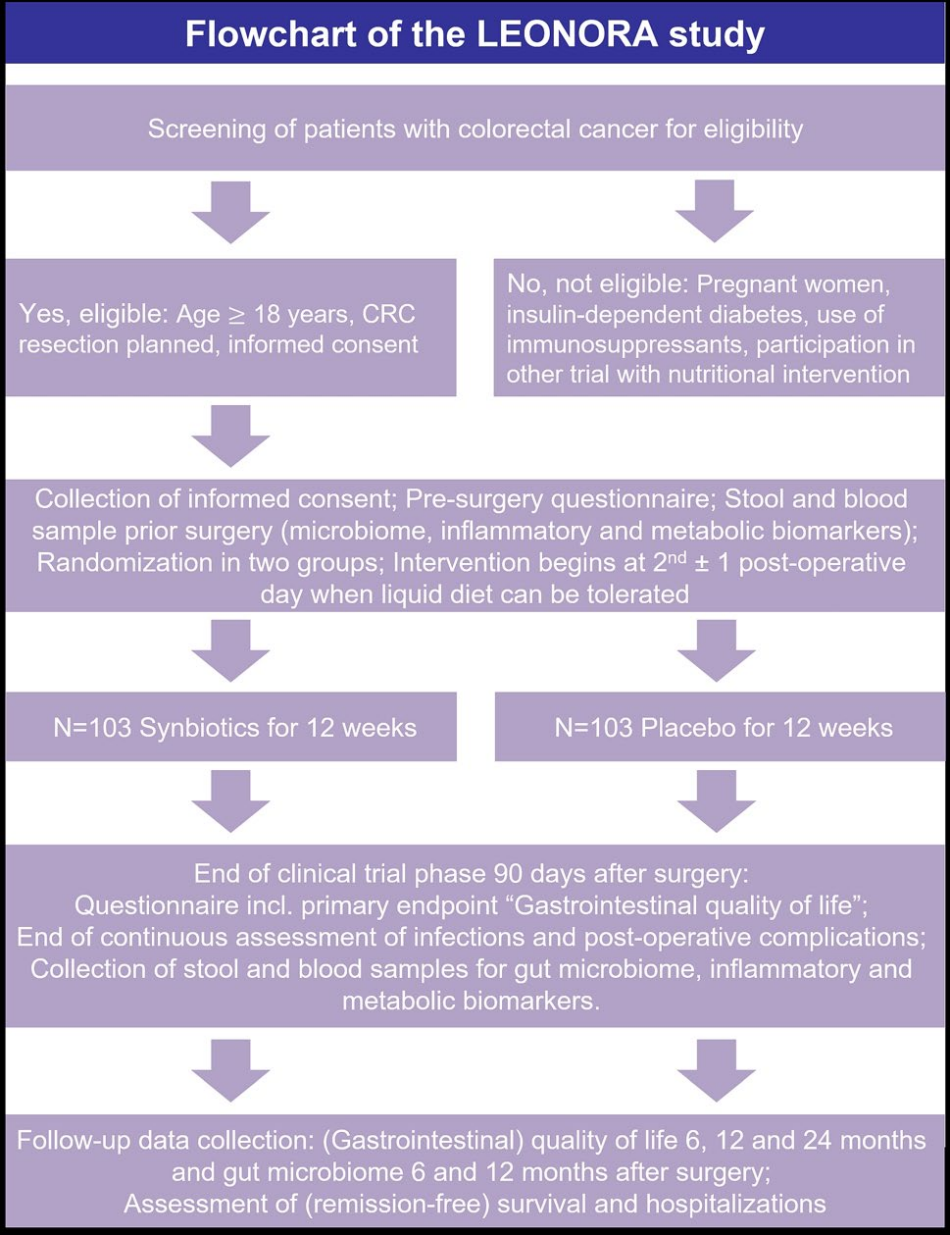
Design of the LEONORA study. Abbreviations: CRC, colorectal cancer

The LEONORA study is a national, multicenter, prospective, double-blinded, randomized, controlled, phase-III trial. Figure 1 shows the schematic flow-chart of the study design. This trial protocol follows the SPIRIT 2025 Checklist for the reporting of protocols of RCTs (Table S2, Supplemental Material).

#### Intervention

The intervention group will receive a commercially available probiotic and prebiotic preparation for daily use for 12 weeks, i.e., 84 days, starting on the first day after surgery, at which oral liquid diet is being tolerated (Day 1–3 post surgery).

The probiotic treatment is Essential-Biotic^®^ Complete delayed-release capsules (Allergy Research Group, CA, USA), which shall be taken once daily. One capsule contains 50 billion colony-forming units (CFU) of 12 different bacterial strains from the Bifidobacterium, and Lactobacillus families, as well as Streptococcus thermophiles.

Multiple-strain probiotics have been suggested to be more effective than single-strain probiotics [22]. The amount of 50 billion CFU probiotic cells is in the middle of the used probiotics of previous trials, which ranged from 3 to 100 billion CFU in most studies [23]. The current literature provides no evidence of an increased risk of complications with probiotics supplementation in humans [6].

The prebiotic treatment is 10 g/day of the fermentable fiber inulin made from chicory plants (Orafti^®^ Synergy1 powder, BENEO GmbH, Mannheim, Germany). This dose is the median dose of 10 previous trials using inulin as part of a synbiotic and administered to patients after oncologic surgery [23]. The coordinating center will fill the daily doses in sachets, whose powder content shall be dissolved in a drink by the study participants. The drink shall be used to swallow the capsule with the probiotic after a meal to reduce the contact with gastric acid.

Flatulence is a common and dose-dependent adverse event of inulin doses ≥ 5 g/day but a tolerance was observed after 2 weeks of use, presumably by microbiota adaptation to the increased fiber availability [24]. To exclude a risk of flatulence during the wound healing phase in the first 4 weeks after surgery, inulin intake will start from week 5 on. The inulin treatment shall start in week 5–6 with 3 g/day, which is the minimal possible dose with a shown effect on microbiota abundance while there is no risk of flatulence, and be continued with 5 g/day in week 7–8 when tolerance towards the very low dose of 3 g is achieved [24]. After intake of 7 g/day in week 9–10, the full dose of 10 g/day shall be applied in week 11–12. The chosen prebiotic and probiotic will act together as a synbiotic because inulin is a specific substrate for the bacteria of the Bifidobacterium family and, to a lesser extent, the Lactobacillus family [18, 25].

#### Control

The control group will receive two different placebos. Using a placebo as the comparator is ethically feasible because synbiotics or probiotics are not part of routine care in German hospitals after CRC resection. The placebo for the probiotic capsule with live bacteria will be a capsule in the same appearance filled with Füllstoff DAC, which is mannitol 35 - a sugar with no effects on microbiota. The placebo for the prebiotic inulin will be maltodextrin powder, which is a sugar with no effects on microbiota, which has the same appearance and almost the same weight, sweet taste, and solubility in water as inulin. The maltodextrin powder will be filled in the same sachets as the inulin powder. Most previous trials testing the effects of inulin on microbiota used maltodextrin powder as the placebo and observed no changes in microbiota in the control group [25].

#### Blinding

The Pharmacy of the University Clinic Heidelberg (Pharmacy) will overcapsule the probiotic with neutral capsules and manufacture the placebo capsules for the probiotic. All probiotic verum and probiotic placebo capsule packages will be labeled with randomization numbers (RandomID) by the Pharmacy obtained from the randomization list.

An office assistant in the coordinating center will weigh the prebiotic intervention (inulin powder) and its placebo (Maltodextrin powder), will fill them in sachets, create packages per study participant containing either inulin or placebo sachets, label them accordingly, and bring them to the Pharmacy. The Pharmacy will label these packages with the randomization numbers (RandomID) and will remove other labels, which could lead to the identification of the randomization group. An office assistant from the coordinating center will fetch the packages for the probiotic/placebo and prebiotic/placebo for each randomization number from the Pharmacy, pack them together, and send these packages to the recruiting study centers. The randomization list will only be kept in the Pharmacy until the trial and statistical analysis are completed. Thus, a triple blind study will be conducted because the study participants, the study physicians/data collectors in the coordinating center, and the trial statistician will not know which study participant received verum and which received placebo.

In case of the need for emergency unblinding (only situations in which it is medically necessary to know the patient’s treatment), the study physician can open a sealed envelope with information about the status (verum or placebo) of the study participant, which will be stored at the study center. Any emergency unblinding must be documented in the eCRF and has to be reported without delay to the coordinating center by the study physician. As such, blinding will remain intact for all other study participants.

#### Randomization

Computer-generated randomization lists to randomize the study participants 1:1 to the intervention and control group will be prepared by the Pharmacy, which will be stratified by recruiting center and cancer location (colon or rectum) using blocks of 2 randomization numbers (RandomIDs) per package, which always contain 1 verum and 1 placebo. The RandomIDs will start with either the letter “K” for colon or “R” for rectum cancer patients and end with a number between 1 and 199 (i.e., K001-K199 and R001-199). If a patient has both colon and rectum cancer, he or she will get a RandomID with a “K”. The coordination center will take care and document that always a block of 2 packages per cancer entity (e.g., RandomIDs K001 and K002 or RandomIDs R001 and R002) are distributed to one center. The RandomIDs will be used to label the study drug packages, informed consent forms, questionnaires, blood sampling kits, stool sampling devices, and all other study materials to be send to the recruiting centers.

#### Study centers

The study will be conducted on a national multicenter basis. The coordinating center is the Division of Clinical Epidemiology of Early Cancer Detection, German Cancer Research Center, Heidelberg. Recruitment is planned to be conducted within 2–3 years in 10 German hospitals in the following towns: Cologne, Heidelberg, Berlin, Essen, Augsburg, Tübingen, Gießen, Stuttgart, Erlangen, and Frankfurt am Main (see Supplemental Material for the institution’s names). The coordinating center will create packages with all study materials and trial drugs needed for a study participant, which are all labelled with the randomization ID (RandomID), which will be supplied by the Pharmacy of the University Clinic Heidelberg, which has the randomization list. The study centers will assign these RandomIDs consecutively to the patients in the timely order of study inclusion and document the RandomID together with the study participants’ name and date of birth in the eCRF.

### Study population

Inclusion and exclusion criteria are listed in Table 1.

**Table 1:** Inclusion and exclusion criteria

Inclusion Criteria	Exclusion Criteria
– Age ≥ 18 years – Diagnosis of CRC (ICD-10: C18-C20; any stage) – Planned CRC resection in one of the up to 12 cooperating German hospitals within the next 14 days – Willingness to complete the pre-surgery questionnaire and to provide the first blood and stool sample or rectal swab before the surgery – Informed consent to participate in the LEONORA trial	– Patient with any social or logistical condition, which in the opinion of the investigator may interfere with the conduct of the study, such as incapacity to understand well, not willing to collaborate, or cannot easily be contacted after discharge – Previous participation in this study – Parallel participation in another interventional trial with a nutritional intervention – Pregnant women – Use of immunosuppressants (Systemic glucocorticoids with a daily dosage of more than 10 mg prednisolone (or equivalent) or drugs of ATC group L04A, except neoadjuvant or adjuvant chemotherapy or radiotherapy for CRC treatment). – Insulin-dependent diabetes – Withdrawn consent prior use of trial medication

*Abbreviations:* *CRC* colorectal cancer, *ICD* International Classification of Diseases, *ATC* Anatomical Therapeutical Chemical classification

### Study endpoints

The primary endpoint of the study is the gastrointestinal function-related quality of life 90 days after surgery, as assessed by the Gastrointestinal Quality of Life Index (GIQLI) total score [26].

Secondary endpoints ascertained during interventional study phase.

- GIQLI total score 30 days after surgery.
- GIQLI subscales 30 and 90 days after surgery: symptoms (e.g. diarrhea, flatulence and constipation), physical functions, emotions and social function subscale.
- Changes in GIQLI total score and sub-scales from pre-surgical visit to day 30 and 90.
- Low Anterior Resection Syndrome (LARS) Score at day 90 after surgery.
- Further quality of life total and sub-scales (EQ-5D-5 L, EORTC QLQ-C30, and EORTC QLQ-CR29) 30 and 90 days after surgery and their changes from pre-surgical visit to day 30 and 90.
- Fatigue scale (EORTC-FA12) and sub-scales at day 90 after surgery.
- Anxiety (GAD-7) at day 90 after surgery.
- Cost-effectiveness (costs per prevented diarrhea case) and cost-utility of the intervention (costs per quality adjusted life-year (QALY) gained, as assessed with the EQ-5D-5 L).
- Emergence of multidrug-resistant organisms (MDRO) until day 90.
- Rate of physician-documented infections (bloodstream infection (BSI), catheter-related BSI, pneumonia, surgical site infections, intra-abdominal infections, and urinary-tract infections) until day 30 and day 90.
- Rate of self-reported infections (upper and lower respiratory tract infections, gastro-intestinal tract infections, bladder infections, wound infections, other infections and episodes with high fever (≥ 38 °C)) until day 30 and day 90.
- Total rate of infection until day 30 and day 90.
- Antibiotics use (Yes/No and number of days) until day 30 and day 90.
- Rates of post-operative complications, defined as the following 4 clinical events: Initiation of antibiotic or antifungal treatment, surgical revision, new mechanical ventilation, sepsis or death until day 90.
- Time from CRC surgery to hospital discharge.
- Number and length of re-hospitalizations until day 90.
- Changes in the gut microbiome composition until day 30 and day 90.
- Concentration changes of biomarkers of inflammation and immune function in serum samples collected prior surgery and 90 days after surgery (89 proteins of the combination of the OLINK^®^ Target 48 Cytokine panel and the OLINK^®^ Target 48 Immune Surveillance panel, including, for example, interleukin-6 as well as biomarkers of systemic inflammatory response (C-reactive protein (CRP), mGPS (modified Glasgow prognostic score), HS_mGPS (high-sensitive mGPS), NLR (neutrophil-to-lymphocyte ratio), PLR (platelet-to-lymphocyte ratio), LMR (lymphocyte-to-monocyte ratio), SII (systemic immune-inflammation index), PNI (prognostic nutritional index), and NPS (neutrophil-platelet score))).
- Concentration changes of metabolomic biomarkers (pre-defined selection of amino acids, fatty acids, and bile acids measured with high accuracy with targeted LC-MS/MS and the total metabolome measured with untargeted LC-MS/MS, which has lower accuracy) in fecal and plasma samples collected prior surgery and 90 days after surgery.

Secondary endpoints with long-term, observational follow-up data.

- Quality of life total and sub-scales (GIQLI, EQ-5D-5 L, EORTC QLQ-C30, and EORTC QLQ-CR29) 6, 12, and 24 months after surgery and their changes from pre-surgical visit to day month 6, 12, and 24.
- Fatigue (EORTC-FA12 and its subscales) at month 6, 12, and 24.
- Depression (CESD-R and its subscales) at month 6, 12, and 24.
- Rate of self-reported infections (upper and lower respiratory tract infections, gastro-intestinal tract infections, bladder infections, wound infections, other infections and episodes with high fever (≥ 38 °C) until month 6, 12, and 24.
- Antibiotics use (Yes/No and number of days) until month 6, 12, and 24.
- Changes in the gut microbiome composition until month 6 and 12.
- Number and length of re-hospitalizations until month 6, 12, and 24.
- 2-5-year overall survival, disease-free survival, and progression-free survival rate.

### Study visits

Table 2 gives an overview on the study visits and more details follow in text below the table.

**Table 2:** Study visits of the LEONORA study

Study visit	Time point	Intervention(s) / Procedure(s)
Pre-surgery visit	Day − 13 prior surgery to day of surgery	Informed consent, randomization, 1st questionnaire, 1st fecal sample (or rectal swab) and 1st blood sample.
Post-surgery visit	Day 1–3 post surgery	Handing out of trial medication and patient diary. Start of treatment as soon as liquid diet is being tolerated for 12 weeks (84 days).
Continuous monitoring for appearance of clinical events and compliance	Day 1 – Hospital discharge	Screening for clinical events (antibiotic/antifungal treatment, surgical revision, ventilation, sepsis or death), and monitoring of regular intake of study medication daily until hospital discharge. If a clinical event is detected, information will be documented in the eCRF.
Day 30 visit	Day 28–49 post surgery	2nd questionnaire and 2nd fecal sample.
Day 90 visit	Day 87–108 post surgery	End of trial. 3rd questionnaire, 3rd fecal sample, 2nd blood sample, and collection of filled patient diary.
Month 6 follow-up visit	Day 183–218 post surgery	4th questionnaire and 4th fecal sample. Collection of data on hospitalizations, infections, cancer treatments, cancer incidence/re-occurrence and vital status at authorities and/or general practitioners/oncologists.
Year 1 follow-up visit	Day 365–400 post surgery	5th questionnaire and 5th fecal sample. Collection of data on hospitalizations, infections, cancer treatments, cancer incidence/re-occurrence and vital status at authorities and/or general practitioners/oncologists.
Year 2 follow-up visit	Day 730–765 surgery	6th questionnaire. Collection of data on hospitalizations, infections, cancer treatments, cancer incidence/re-occurrence and vital status at authorities and/or general practitioners/oncologists.

*Abbreviations:* *eCRF* electronic Case Report Form

#### Screening

Screening for eligible study participants will be done by a study nurse or study physician in the patient’s files (which captures clinical diagnoses) before or shortly after their admission to the hospital with a standardized screening checklist stating the in-and exclusion criteria.

#### Pre-surgery visit for information about the study (Day − 13 prior surgery to day of surgery)

During a first appointment in the hospital, eligible patients will be informed about the LEONORA study and the secondary data use in the National Center for Tumor Diseases (NCT) network by a study physician and will receive the printed study information documents. Written informed consent to participate in the LEONORA study will be obtained.

A study nurse or study physician will pick the baseline questionnaire from a study package, which is labelled with the RandomID, and give it to the study participant who shall fill it on the same day while being in the clinic. It is important that the study nurse takes a package with a RandomID starting with the letter “K” for a colon cancer patient or with a RandomID starting with the letter “R” for a rectum cancer patient. If a patient has both colon and rectum cancer, a package with a RandomID starting with a “K” shall be picked.

The study nurse or study physician will collect the baseline questionnaire, check it for completeness and will ask the study participant to complete the missing items immediately. The questionnaire will collect important data on the baseline levels of the quality of life endpoint scales and information needed to be able to contact the study participants for the follow-ups (name, sex, birthday, address and phone number).

Furthermore, the first stool sample will be collected on the Day − 13 to Day − 1 prior, with swabs that are labelled with the RandomID. If study participants cannot donate a stool sample in the given time frame, they will be asked to collect a rectal swab. The fecal sample should be obtained prior to the initiation of laxative treatment if performed pre-operative as part of routine patient management standards. Furthermore, 27 ml blood will be taken from each patient on the same day using tubes labelled with the RandomID. If possible, the blood sampling will be included in a regular blood sampling that is needed anyway for the routine patient care.

#### Post-surgery visit (Day 1–3 post surgery)

As soon as liquid diet is being tolerated (1–3 days after surgery), the trial nurse/physician will hand out the trial medication and the patient diary and explain their use to the patient. Both are labelled with the trial participants randomization ID. The day of the surgery is day 0 of the trial. The interventional phase starts with intake of the trial medication, which will last 12 weeks (84 days). The study participants are being asked to document intake of the trial drugs as well as all symptoms, new diagnosed diseases and injuries, which occurred during these 84 days, in the diary.

Continuous monitoring for appearance of clinical events and compliance (Day 1 – hospital discharge).

• Check for compliance and occurrence of a clinical event daily after surgery and until discharge. A clinical event refers to the occurrence of one of the 4 clinical incidents defined below.

- ◦ Treatment: Initiation of antibiotic or antifungal treatment excluding perioperative or other prophylaxis.
- ◦ Revision: Surgical revision or deep puncture / biopsy and/or interventional drainage (during endoscopic or percutaneous procedures) for any cause and any other consecutive abdominal surgery including repositioning of stoma.
- ◦ Sepsis
- ◦ Ventilation: New intubation and mechanical ventilation for any reason OR prolonged ventilation (≥ 24 h following surgery).

- For each patient a maximum of three distinct clinical events is assessed. The first clinical event is triggered by the appearance of at least one of the above defined clinical incidents and may include further clinical incidents within 72 h (e.g. initiation of antibiotic treatment and intubation).
- If a clinical event occurs, it will be documented in detail in the eCRF.
- Readmission and occurrence of clinical events after the initial inpatient stay will be assessed by the patient diary, which will be filled until the Day 90 visit, and the Day 90 questionnaire. Medical records of patients with clinical events, including discharge letters, will be retrieved from the patients’ treating physicians by the coordinating center if deemed necessary for complete documentation.

#### Day 30 visit (Day 28–49 post surgery)

The 2nd questionnaire and the 2nd fecal sample collection kit will be mailed to the study participants by the coordinating center 4 weeks after the surgery (Day 28). The study participants are being asked to send back the filled questionnaire and the stool sample to the coordinating center by mail in a prepaid return envelope.

#### Day 90 visit (Day 87–108 post surgery)

The 3rd questionnaire and fecal sample collection kit will be mailed to the study participants by the coordinating center on Day 87 post-surgery, which is the last possible day trial drugs could be used (84 days of treatment + start up to 3 days after surgery). The study participants are being asked to send back the filled questionnaire and the stool sample to the coordinating center by mail in a prepaid return envelope. In addition, the study participants will be asked to send the patient diary to the coordinating center with a prepaid return envelope. Furthermore, they will be asked to donate the 2nd blood sample (38.6 ml). Depending on the distance between the patient’s home and the clinic that recruited them, they will be asked to donate this blood sample either in the clinic or to their general practitioner. In the latter case, all necessary materials will be sent from the coordinating center to them by mail.

#### Month 6 (Day 183–218), year 1 (Day 365–400), and year 2 (Day 730–765) follow-up visits

At these times, staff of the coordinating center will send the study participants the 4th, 5th, and 6th questionnaires. Furthermore, the 4th and 5th fecal sample collection will take place at the month 6 and year 1 visit in the same way as in the visits before.

Furthermore, the coordinating center will contact study participants’ general practitioners and/or oncologists in order to obtain medical records about cancer treatments and infections after release from the hospital and the course of the treatment (incl. re-hospitalizations and cancer recurrence). At the same time, if the staff of the coordinating center was unable to contact a study participant by telephone or mail, the authorities (residents’ registration office, registry office, and/or health authority) will be contacted to find out about the current address and vital status (cause of death, if applicable). If establishing a phone contact with a study participant is difficult, filling out the questionnaires can take place up to 6 months after the planned date.

As the availability of information at authorities about the vital status and cause of death can be delayed by up to 2 years, and the recruitment time will be 2–3 years, we will store the personal data needed for these inquiries for a minimum of 6 years. The obtained overall, cancer, and CRC-specific survival time will vary from 2 to 5 years for the study participants because the mortality follow-up will end at a fixed date (31 Dec 2030). This implies that the last recruited study participant will have the shortest observation period of approx. 2 years, and the first recruited study participant will have the longest observation period of approx. 5 years.

### Data management

#### Electronic case reporting file

For extensive clinical data collection, an eCRF using the ClinicalSurveys.net online platform of the University Hospital of Cologne will be used. ClinicalSurveys.net is hosted by QuestBack, Oslo, Norway, on servers in Cologne, Germany, as part of a software-as-a-service agreement. The proprietary software allows rapid design and deployment of electronic CRFs. Study personnel log in to the system with a username and a safe password including letters, numbers, and symbols. Study personnel can only view and modify their own contributions. All data transmissions are encrypted via TLS 1.2 with an AES 256 GCM bit key and ECDHE RSA key exchange; certificate provided by COMODO RSA Domain Validation Server. Data for the LEONORA study is only documented pseudonymously; no directly identifying data are stored on QuestBack servers.

Administration of the eCRF is limited to selected and named administrators at the University Hospital of Cologne and the coordinating center at the DKFZ, who receive comprehensive training in the system before access is granted. Secure passwords are also enforced for administrators, and they have to regularly change their passwords. Any data manipulation by users and administrators is logged in an audit trail allowing complete data reconstruction. Server administration is performed by QuestBack, and includes regular updates of the linux-based servers, rigid firewall configuration, current virus and threat detection, and daily backups (on-site and off-site with secure storage). Regular on-site audits of security and data protection measures are performed at QuestBack Cologne by the University Hospital of Cologne.

Each study personnel will have specific access and rights defined by their respective role. The system will fulfil standards of Good Epidemiological Practice with state-of-the-art security and data protection standards. Data will be entered accurately and comprehensively by the responsible site personnel. Central and partly automatic monitoring will be performed for plausibility and completeness of data and queries may be issued to the site in case of ambiguity or fault. Data documentation should be performed continuously and at the latest shortly (1 month) after completion of each visit. Data with regard to long-term outcomes will be documented at the respective timepoint.

On-site monitoring of documentation will not be generally conducted at all study sites. However, in case of considerable doubt regarding compliance to study protocol adherence, documentation quality or accuracy, the study coordinators may perform on-site source data monitoring using qualified personnel. During such audits, entries in the eCRF will be compared with the original source documents. The site leader will cooperate in resolving any queries or findings made during the whole monitoring process. A data monitoring committee will not be initiated because the data management team in the University Clinic Cologne, in charge of the monitoring, has enough experienced from previous studies.

The eCRF will be used to collect the following data:

- General Information: Study-ID, Age, Sex, weight, height, Inclusion/Exclusion Criteria.
- CRC information (stage prior surgery, location (ICD-10 code)).
- Information on patient status (comorbidities, concomitant medication, oncological treatment regimen (chemotherapy incl. number of cycles/ radiotherapy), current location (inpatient, department, ward), known allergies against anti-infective substances, admission, readmission).
- Information on surgical procedure.
- Information on clinical events.
- Detection of MDRO during any local microbiological sampling.
- Occurrence of consecutive surgeries, including possible repositioning of stoma.
- Data collected at the study visits conducted while study participants are hospitalized with exception of the questionnaire data.
- Concomitant medication.
- Anti-infective treatments.

#### Questionnaire data

Patients will undergo a structured assessment of their quality of life at 6 different time points throughout the study period. Assessment will be performed using the standardized GIQLI, EQ-5D-5 L, EORTC QLQ-C30 core questionnaire and EORTC QLQ-CR29 module [26–29].

The pre-surgery visit questionnaire additionally contains questions about socio-demographic factors and frailty (FRAIL scale [30]). To keep this first questionnaire short, questions on school education, social network (Duke-UNC FSSQ-5 ([31]), co-morbidity, time since the CRC diagnosis, smoking history, and family history of CRC were added to the day 30 questionnaire.

In the day 90, month 6 and month 12 questionnaires, lifestyle questionnaires were added (smoking, alcohol consumption, physical activity, nutrition as well as height and weight for body mass index calculation) using standardized instruments (HEALTHY life-style score [32], Rapid Assessment of Physical Activity questionnaire (RAPA) [33], and the FiberScreen 18-item food frequency questionnaire to assess the amount of fiber in the diet [34]).

Furthermore, questions related to cancer treatments (chemotherapy, radiotherapy, additional surgery), hospitalizations (dates, length and reason), infections (number, location, vaccinations), antibiotics use, and newly developed cancers or CRC reoccurrence (location, date) were added to all follow-up questionnaires from day 90 on.

The questionnaires will be scanned, the scanned information will be verified by visual inspection, and the data will be electronically stored in pseudonymized manner on secured servers in the coordinating center. Scanned information from the pre-surgery visit, day 30, and day 90 questionnaire, needed for the primary and secondary endpoint(s) during the interventional phase of the study, will be verified by two independent documentarists and disagreements will be resolved by inspection of the original questionnaire.

#### Patient diary

The study participants are being asked to document intake of the trial drugs as well as all symptoms, new diagnosed diseases and injuries, which occurred during the 84 days of trial drug intake, in the diary. If compliance with drug intake is unclear, the study participants will be called by phone. If compliance remains unclear (e.g., if study participant cannot be reached), incompliance will be assumed. If the study participants consent to receive SMS massages from the coordinating center and provide their mobile number, they will receive one SMS per week as a short reminder to take the trial drugs and complete the patient diary information for this week.

No trial drug discontinuing criteria, e.g., based on potential adverse events, are stated in the study protocol. As treatment intake is voluntary, the study participants can discontinue treatment at any time, and should document this in the patient diary.

### Biological material

#### Sample collection

Table 3 shows the collected biological specimen at the specific study visits.

**Table 3:** Sample collection

Study Visit	Blood samples	Stool suspension samples
Pre-surgery visit	• 1 × 9 ml EDTA tube • 2 × 9 ml Serum tube	• 1 ml COPAN eNAT™ ^a^ • 1 ml COPAN eSwab™ ^a^
Day 30 visit	**-**	• 1 ml COPAN eNAT™ • 1 ml COPAN eSwab™
Day 90 visit	• 1 × 9 ml EDTA tube • 1 × 2.6 ml EDTA tube • 3 × 9 ml serum tube	• 1 ml COPAN eNAT™ • 1 ml COPAN eSwab™
Month 6 follow-up visit	**-**	• 1 ml COPAN eNAT™
Month 12 follow-up visit	**-**	• 1 ml COPAN eNAT™

^a^If no stool sample collection was possible at the pre-surgery visit, rectal swabs will be collected with the same devices

#### Sample Storage

All study sites will collect, process, and store samples in local freezer storage at temperatures between − 32 °C to -80 °C in boxes or crates. The samples will be sent to the laboratory of the coordinating center in batches every 6 months. All samples can be stored for up to 30 years and must then be destroyed or disposed.

#### Data and biospecimen use in the biobank of the NCT network

Surplus biospecimen (blood and stool samples) not needed for the purposes of the LEONORA study will be transferred to the biobank of the NCT network for those study participants who provided additional informed consent for participation in this biobank. As outlined in the patient information and consent form for this biobank, they will be used for secondary medical research purposes. The aim of research in the NCT network is to understand the causes and mechanisms of the development and progression of cancer, to quickly transfer promising approaches from cancer research to the clinic and thus benefit future patients. Prior to any data access, a project with data of biobank of the NCT network needs to be approved by an ethics committee. Scientists of the NCTs and collaboration partners are allowed to ask for data access for a specific project with an ethic committee approval at the Use and Access Committee of the biobank of the NCT network. If approved, data and biospecimen will be shared in pseudonymized form.

### Biomarker measurements

#### Cultural microbiology

For detection of MDRO and recultivation of pathogens of interest, samples will be sent to the University Hospital of Cologne. Samples obtained during study conduct will be subjected to a targeted culture for detection of MDRO and pathogens of interest (Enterobacterales, non-fermenting bacteria, Staphylococcus aureus). This needs to be assessed for every included patient in the samples from day 90, and, depending on occurrence of study events after day 90 should be repeated in later samples. Samples will be subjected to selective culture using suitable media (i.e. chromagar) and subsequent isolate characterization including antimicrobial resistance testing and PCR for selected resistance genes if applicable.

In cases with detection of MDRO colonization in the day 90 samples, baseline and day 30 samples will be selected for cultural analysis in order to differentiate between newly acquired and preexisting MDRO colonization.

If there is not yet clarity about the causative pathogen, samples preceding the development of an infection may be selected to recultivate the causative pathogen from its likely endogenous origin (i.e. fecal sample). Identified bacteria will be subjected to antimicrobial resistance testing and may be selected for further characterization via whole genome sequencing.

#### Whole genome-sequencing of selected isolates

Selected isolates both from study samples and infection-related samples might be subjected to whole genome sequencing. Briefly, sequencing libraries and sequencing might be performed using state of the art library preparation kits and sequencers (Illumina) followed by de novo assembly using suitable software (i.e. Velvet). Assembled genomes may then be used for comparison i.e. using cgMLST and traditional 7-loci MLST using SeqSphere+ software version 6.0.2 (Ridom, Münster, Germany). Furthermore, presence of resistance genes will be determined.

#### Microbiota analysis

Microbiota analysis of selected samples will be performed at the central laboratories of University Hospital Tübingen for fecal samples. Briefly, genomic DNA will be extracted from samples using suitable extraction kits (i.e. FastDNA Spin Kit for Soil for fecal samples, Biomedicals, Solon, OH, USA). Concentration and quality of the extracted DNA will be assessed spectrophotometrically (Nanodrop One, Thermo Fisher Scientific, Waltham, MA, USA) and the amount of double-stranded DNA will be quantified using the Qubit dsDNA kit (Thermo Fisher Scientific, Waltham, MA, USA). Libraries for shotgun metagenomic sequencing will be prepared using suitable protocols (e.g. adaptor-ligation using the NEXNext Ultra II FS DNA Library Prep kit and NEBNext Multiplex Oligos for Illumina (New England Bioabs, Ipswich, MA, USA)), followed by sequencing using the NovaSeq 6000 platform (Illumina, San Diego, CA, USA) to allow for cost efficient ultra-deep sequencing of the samples (up to 8.2B 2 × 150 base pairs per sample/ 5.9 Gb per sample). In selected cases, sequencing may be additionally performed on a long-read sequencing platform (i.e. PromethIon).

For data analysis, suitable bioinformatics platforms and approaches will be applied to achieve taxonomic classification (i.e. Kraken2, Kaiju, DIAMOND/MEGAN) and functional pathway profiling (i.e. using HUMAnN 2.0).

#### OLINK^®^ Target 48 Cytokine and Target 48 Immune Surveillance panels

Staff of the laboratory of the Department of Clinical Epidemiology of Early Cancer Detection of the German Cancer Research Center (DKFZ) will pipet 10 µL serum on plates provided by the company OLINK, Uppsala, Sweden. The plates will be used to measure the OLINK^®^ Target 48 Cytokine and Target 48 Immune Surveillance in the core facility of the DKFZ, which will lead to the measurement of 89 unique proteins with absolute quantification, which are relevant for immune function and inflammation.

#### Metabolomics

A pre-defined set of metabolomic biomarkers (amino acids, fatty acids, and bile acids) will be measured with targeted Gas or Liquid Chromatography-Tandem Mass Spectrometry (GC-MS/MS or LC-MS/MS) in fecal and plasma samples collected prior surgery and 90 days after surgery at the metabolomics platform of the NCT Dresden, which will ensure high accuracy by using acknowledged standards for the metabolites. Furthermore, an untargeted LC-MS/MS approach will be applied with the same samples at the metabolomics platform of the NCT Dresden, which will lead to a high number of measured metabolites (~ 3,000) but with lower accuracy because no standards for specific metabolites will be used.

### Statistical analysis plan

Homogeneity of the intervention and control groups will be described by comparison of the demographic data and key baseline characteristics. All statistical tests will have a two-sided significance level of 0.05. In addition, 95% confidence intervals will be estimated for all outcomes in the intervention and control groups. All analyses will be done using SAS software version 9.4 or later.

The primary analysis will test the null hypothesis:

- H0:GIQLI total score 90 days after surgery is not different in the two groups. versus the alternative hypothesis:
- H1: GIQLI total score 90 days after surgery is different in the two groups.

The GIQLI total score at day 90 can be assumed to be normally distributed. A linear regression model adjusted for study center and the baseline GIQLI total score will be used to test the null hypothesis. Multiple imputation will be applied to impute missing covariate data only.

The primary endpoint GIQLI total score will be analyzed with a modified intention-to-treat (mITT) approach, including all randomized patients, but excluding patients who did not start with the trial medication (retroactively excluded patients).

Reasons for primary endpoint data to be missing are a lack of interest in filling the questionnaire, or therapy (chemo and/or radiotherapy) between day 90 and day 108 (which hinders patients from answering the 90-day questionnaire), or staying in the rehabilitation clinic between day 90 and day 108 (which makes them hard to reach). With clinical and treatment information collected up to day 90 via inquiry at the general practitioners and oncologists treating the patients in the outpatient setting, and the baseline data GIQLI value, age, sex, education, pre-operative cancer stage, and tumor location (left colon / right colon/rectum), missing 90-day GIQLI data can be considered missing at random. Consequently, a sensitivity analysis will be performed with multiple imputed 90-day GIQLI values. Multiple imputation will be performed separately for each arm. For study participants who withdrew consent and requested deletion of all data, mean imputation will be used. Study participants who withdrew consent prior first use of trial medication will also be excluded from this sensitivity analysis (mIIT).

A per-protocol analysis will be done additionally as a sensitivity analysis, excluding study participants who fulfill one or more of the following protocol deviations:

- Incompliance: Defined as intake of trial drugs on less than 80% of the treatment days (i.e., < 67 days) as documented in the patient diary. If no patient diary was sent back to the coordinating center, this also counts as incompliance.
- Late completion of the day 90 questionnaire: Defined as > 115 days after surgery.
- Use of probiotic product in addition to the study medication (i.e., any time between post-surgery and day 90 visit).
- Late noticed false inclusion in the trial (inclusion criteria not fulfilled or exclusion criterion was present).

All secondary endpoints with a continuous scale will be analyzed with the same statistical methods as described for the primary endpoint above, with a linear regression model adjusting for center and the baseline value of the endpoint (if accessed). The dichotomous secondary endpoints will be tested with a logistic regression model or Cox proportional hazards regression model (depending on the nature of the endpoint) adjusted for center. In addition, a generalized estimating equation (GEE) model will be applied for all outcomes that have been repeatedly assessed.

The following a priori defined subgroup analyses will be conducted (The definition of pre-operative is “Assessed at pre-surgery visit,” and the definition of peri-operative is “Ascertained between pre-surgery visit and day 90 visit”):

- Age (< 65 / ≥ 65 years).
- Sex (female/male).
- Pre-operative cancer stage (I or II / III / IV).
- Tumor location (left colon / right colon/rectum).
- Pre-operative frailty (Non-frail / pre-frail / frail, as assessed with FRAIL scale [27])
- Post-operative stoma (no/yes, on day 30 only / yes, on day 30 and 90).
- Peri-operative antibiotic treatment (no (except single shot) / yes (additional to single shot)).
- Long-term post-operative antibiotic treatment (< 10 / ≥ 10 days).
- Adjuvant chemotherapy and/or radiotherapy (no/yes).
- Post-operative chemotherapy (no/yes, but not in week prior day 90 questionnaire date / yes, and in week prior day 90 questionnaire date).
- Post-operative radiotherapy (no / yes, but not in the week prior to the day 90 questionnaire date / yes, and in the week prior to the day 90 questionnaire date).
- Pre-operative GIQLI score (≤ median / > median).
- Pre-operative physical activity (≤ median / > median recorded with the Rapid Assessment of Physical Activity questionnaire (RAPA) [33]).
- Pre-operative alcohol consumption (Abstinent/ Alcohol consumption ≤ median / Alcohol consumption > median by grams of ethanol per week).
- Pre-operative smoking (current smoker / current non-smoker).
- Pre-operative Healthy Lifestyle Score (0–2 / 3–5 points [32])
- Pre-operative BMI (< 23 / 23–30 / > 30 kg/m²).
- Pre-operative cachexia (yes/no, definition of Blum et al. based on current BMI and weight loss in the last 6 months [35])
- Weight change from pre-surgery visit to day 90 questionnaire (≤ median / > median ∆BMIpct).
- Post-operative dietary fiber intake (≤ median / > median recorded with the validated FiberScreen 18-item food frequency questionnaire at day 90 [34])

Because of the multiplicity of tests for subgroup and secondary outcome analyses, all results except for the primary endpoint will be regarded as exploratory and not confirmatory.

No interim analyses are planned.

### Sample size estimation

The sample size estimation is based on the primary outcome, GIQLI total score, which was assumed to be normally distributed. The expected mean (SD) GIQLI total score in the synbiotics group (77.0 (9.9)) and placebo group (72.5 (9.9)) were the values reached after 3 months in the comparable trial of Theodoropoulos et al. [17]. With a significance level of 0.05 and 80% power, 154 patients (77 per group) are needed to detect a relevant score difference using a two-sample t-test for the mean difference (SAS Institute Inc., Cary, NC, USA, PROC Power). The number of patients to be randomized (*N* = 206) was calculated with an assumed drop-out rate of 25% until trial day 90 (*N* = 154 to be analyzed). A dropout is defined by the unavailability of data for the primary outcome (GIQLI total score at trial day 90).

## Discussion

### Expected benefits

The intestinal microbiome is often severely impaired after colon cancer surgery, especially if additional antibiotic therapy is required, which is particularly evident in a reduction of important and beneficial bacteria. The synbiotic could help the gut microbiome recover more quickly and increase the diversity of microorganisms. This, in turn, could lead to a reduced burden of diarrhea and bloating. However, as the effects of this symbiotic on the gut microbiome of CRC patients have not yet been tested, it is also possible that no changes will result from the participation in this study.

The placebo group will have no personal benefit from participating in the study despite the “placebo effect”. However, by participating in the LEONORA study, they are making an important contribution to scientific research. The results of the study may help to determine the effects of the synbiotic on the disturbed intestinal microbiome and the quality of life after colon or rectum cancer surgery, so that future patients may be able to benefit from this synbiotic.

### Possible harms

No adverse events have been reported in connection with the intake of probiotics, both in healthy people and in patients with a wide range of illnesses, including. CRC [6]. Furthermore, no interactions with medication or other medical or therapeutic measures are known.

With respect to prebiotics, with the exception of temporary flatulence and a bloated stomach, which can occur when starting to take dietary fibers such as inulin, no adverse events are known. Flatulence is a common and dose-dependent adverse event of inulin doses ≥ 5 g/day but a tolerance was observed after 2 weeks of use, presumably by microbiota adaptation to the increased fiber availability [24]. In the LEONORA trial, the inulin treatment shall start in week 5–6 with 3 g/day, which is the minimal possible dose with a shown effect on microbiota abundance while there is no risk of flatulence, and be continued with 5 g/day in week 7–8 when tolerance towards the very low dose of 3 g is achieved [24]. After intake of 7 g/day in week 9–10, the full dose of 10 g/day shall be applied in week 11–12. Thus, there are always 2 weeks before a dose increase, in which the gut microbiota can adapt to the increased availability of inulin.

As no serious adverse events related to the trial medication are expected, no ancillary or post-trial care measures are planned.

### Benefit-to-harm ratio

In summary, we assume that the expected benefits of the synbiotic treatment will outweigh the possible harms.

### Patient involvement

The patient representative, Michael Butler, read the synopsis of the trial protocol and commented specifically on the inclusion and exclusion criteria as well as the endpoints of the trial. Michael Butler supports the concept of the trial entirely and, starting in October 2023, agreed to give advice to the study’s PIs as needed throughout the preparation time and while the trial is running. The trial’s principal investigators will ensure interactive patient involvement as part of the study in order to support the study in various aspects. For example, Michael Butler commented on the study’s patient information, consent forms, questionnaire, and flyer, and he will be included in the study’s Steering Committee to provide valuable feedback in meetings from a patient’s perspective while the trial is running. Also, Michael Butler participates regularly in the bi-weekly LEONORA Organization Meeting of the study’s PI. When the final results are available, the dissemination strategy will be drafted interactively with the patient representative to use his knowledge about outreach possibilities to CRC patients and their advocacy groups as much as possible.

### Dissemination plan

All results of the trial will be published in international, peer-reviewed journals and in the trial registry DRKS (https://www.drks.de/search/de/trial/DRKS00034919). Furthermore, the results will be summarized in lay language for the general public on the homepage of the Division of Clinical Epidemiology of Early Cancer Detection at the German Cancer Research Center (https://www.dkfz.de/en/clinical-epidemiology-of-early-cancer-detection/leonora). The outreach to patient advocacy groups will be planned with the patient representative when the trial results are obtained.

### Trial status

The first trial participant was included in September 2025.

## Supplementary Information

Supplementary Material 1.

## Data Availability

The data will not be published on an Open Access Platform. After completion of the study, interested scientists can request data use and receive pseudonymized data upon approval of this application by the principal investigators of the trial (Ben Schöttker and Lena Biehl).

## Abbreviations

ATC: Anatomical Therapeutical Chemical Classification
BSI: Bloodstream Infection
CESD-R: Center for Epidemiologic Studies Depression Scale Revised
CFU: Colony Forming Units
CRP: C-reactive protein
DKFZ: German Cancer Research Center (Deutsches Krebsforschungszentrum)
DRKS: Clinical Trials Register (Deutsches Register Klinischer Studien)
eCRF: electronic Case Report Form
EDC: Electronical Data Capture
EORTC QLQ-C30: European Organization for Research and Treatment of Cancer – core Quality of life questionnaire with 30 items
EORTC-QLQ-CR29: European Organization for Research and Treatment of Cancer – Colorectal cancer
EORTC-FA12: European Organization for Research and Treatment of Cancer – Cancer related fatigue
EQ-5D-5L: EuroQol 5 dimensions 5-level
GAD-7: Generalized Anxiety Disorder Scale
GC-MS/MS: Gas Chromatography-Tandem Mass Spectrometry
GEE: Generalized Estimating Equation
GIQLI: Gastrointestinal Quality of Life Index
GP: General Practitioner
HS_mGPS: High-Sensitive Modified Glasgow Prognostic Score
ICD: International Classification of Diseases
ICTRP: International Trials Registry Platform
ITT: Intention-To-Treat
LARS: Low Anterior Resection Syndrome
LC-MS/MS: Liquid Chromatography-Tandem Mass Spectrometry
LMR: Lymphocyte-To-Monocyte Ratio
MDRO: Multidrug Resistant Organisms
mGPS: Modified Glasgow Prognostic Score
NCT: National Center for Tumor Diseases
NLR: Neutrophil-to-Lymphocyte Ratio
NPS: Neutrophil-Platelet-Score
PLR: Platelet-to-Lymphocyte Ratio
PNI: Prognostic Nutritional Index
QALY: Quality Adjusted Life-Year
RAPA: Rapid Assessment of Physical Activity questionnaire
RCT: Randomized controlled trial
SII: Sytemic Immune-Inflammation Index
SM: Sample Manual
